# Clinical and radiographic improvement in suspected pulmonary lymphangitic carcinomatosis-associated hypoxemic respiratory failure following iruplinalkib-based treatment in ALK-positive lung adenocarcinoma after sequential treatment with ensartinib and lorlatinib: a two-patient case report

**DOI:** 10.3389/fmed.2026.1917214

**Published:** 2026-10-13

**Authors:** Hanbin Lin, Yuxiong Hu, Haoqiang Li, Zhongshan Yang, Ying Zhang, Ruina Luan, Qingyu Lin, Maohe Chen

**Affiliations:** 1 Central Laboratory, Affiliated Hospital of Putian University, Putian, Fujian, China; 2 Department of Respiratory and Critical Care Medicine, Affiliated Hospital of Putian University, Putian, Fujian, China; 3 Department of Thoracic and Cardiovascular Surgery, Affiliated Hospital of Putian University, Putian, Fujian, China; 4 School of Basic Medical Sciences, Key Laboratory of Translational Tumor Medicine in Fujian Province, Putian University, Putian, Fujian, China

**Keywords:** ALK-positive lung adenocarcinoma, hypoxemic respiratory failure, iruplinalkib, lorlatinib, pulmonary lymphangitic carcinomatosis

## Abstract

Pulmonary lymphangitic carcinomatosis (PLC) is an uncommon but life-threatening manifestation of advanced lung cancer and may rapidly lead to hypoxemic respiratory failure. Evidence guiding subsequent anaplastic lymphoma kinase (ALK)-directed therapy after prior exposure to multiple ALK tyrosine kinase inhibitors remains limited, particularly in patients with acute respiratory compromise. In this report, we describe two never-smoking women with stage IVB ALK-positive lung adenocarcinoma who developed type I respiratory failure. This was accompanied by clinical and radiographic findings suggestive of PLC after sequential treatment with ensartinib and lorlatinib. Case 1 achieved a partial response to first-line treatment with ensartinib for approximately 19 months. At progression, plasma next-generation sequencing identified an EML4–ALK variant 1 fusion with an acquired ALK p.L1196M resistance mutation. During subsequent lorlatinib therapy, the patient developed rapidly progressive dyspnea, diffuse bilateral peribronchovascular and reticular opacities suggestive of PLC according to radiographic findings, and type I respiratory failure. Rapid improvement in oxygenation was observed following initiation of third-line iruplinalkib monotherapy. Case 2 had ALK-positive lung adenocarcinoma with an intravascular tumor thrombus on diagnostic biopsy. After ensartinib discontinuation due to grade 3 constipation and subsequent lorlatinib therapy, the patient developed type I respiratory failure, pleural effusion, and progressive bilateral pulmonary abnormalities suggestive of PLC according to radiographic findings. Iruplinalkib was administered in combination with salvage pemetrexed. Following initiation of the respective treatment regimens, both patients showed rapid improvement in oxygenation and radiographic observations of pulmonary abnormalities. Case 1 received iruplinalkib monotherapy, whereas case 2 received iruplinalkib and pemetrexed; therefore, treatment-specific attribution was not possible in case 2. No adverse events attributable to iruplinalkib were observed. In these two patients, initiation of iruplinalkib-based treatment was followed by rapid improvement in clinical and radiographic findings, supporting further evaluation of this treatment strategy in similar critically ill patients.

## Introduction

Anaplastic lymphoma kinase (ALK) rearrangements occur in approximately 3%–7% of non-small cell lung cancers (NSCLCs) and define a subgroup that substantially benefits from ALK tyrosine kinase inhibitors (TKIs) (1). Next-generation ALK TKIs with central nervous system activity are preferred first-line options; after progression, treatment is individualized according to prior TKI exposure, the pattern and tempo of progression, patient fitness, and molecular resistance findings when reassessment is feasible (2, 3). No standard ALK-targeted strategy has been established after disease progression under lorlatinib therapy, which may involve compound ALK mutations, bypass pathway activation, or histologic transformation (4–6). Iruplinalkib is an ALK/ROS1 TKI approved in China with demonstrated activity in earlier treatment settings (7–9), but evidence of its therapeutic effects after treatment with lorlatinib remains limited (10).

Pulmonary lymphangitic carcinomatosis (PLC) is characterized by malignant involvement of the pulmonary lymphatic network and may lead to rapidly progressive dyspnea, hypoxemia, and respiratory failure (11). Clinicoradiological studies have reported that ALK-rearranged NSCLC is more frequently associated with lymphangitic carcinomatosis, advanced nodal disease, pleural effusion, and pleural metastasis than ALK/EGFR-negative NSCLC (12, 13). Previous reports have described improvement in the condition of critically ill or mechanically ventilated patients with ALK-positive NSCLC after treatment with crizotinib or alectinib (14, 15), and a 2023 conference report described a rapid decline in PLC-associated acute respiratory failure following brigatinib treatment (16). However, none of these reports evaluated treatment strategies incorporating iruplinalkib after sequential treatment with ensartinib and lorlatinib.

Against this background, we describe two patients with ALK-positive lung adenocarcinoma who developed severe hypoxemic respiratory failure, with their clinical and radiographic findings suggestive of PLC after prior treatment with ensartinib and lorlatinib. Iruplinalkib-based treatment was initiated, followed by rapid improvement in clinical and radiographic findings. These two cases add clinical experience with iruplinalkib-based treatment after lorlatinib treatment in this underreported, life-threatening clinical setting.

## Case presentation

Written informed consent for publication of the clinical information and accompanying images was obtained from both patients. Their clinical courses and key characteristics are summarized in Figure 1 and Table 1, respectively.

**Figure 1:**
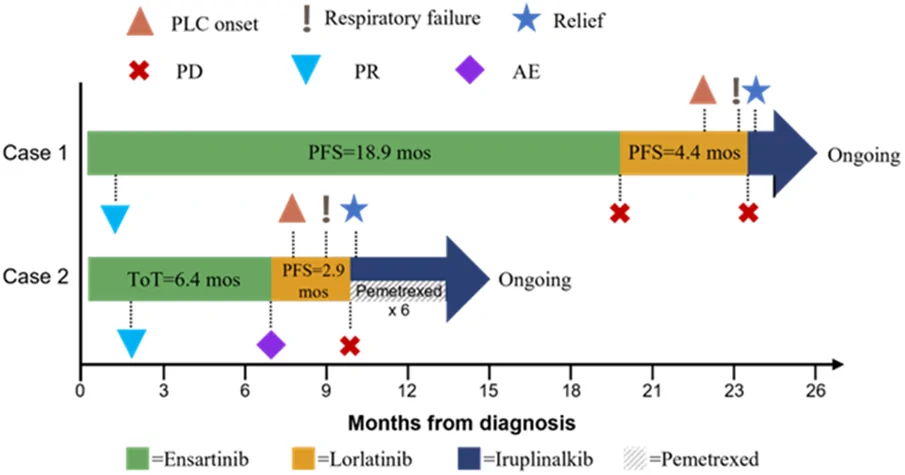
Clinical treatment timelines and disease evolution of the two patients with ALK-positive lung adenocarcinoma. The horizontal axis indicates time in months from the initial diagnosis. The horizontal colored bars represent the sequence and duration of systemic therapies: ensartinib (green), lorlatinib (orange), iruplinalkib (dark blue), and pemetrexed (gray hatched). The geometric symbols indicate key clinical events: the light blue inverted triangles denote partial response (PR), purple diamonds indicate treatment-limiting adverse events (AE), red crosses mark progressive disease (PD), brown upward triangles indicate the onset of clinical and radiographic findings suggestive of PLC, black exclamation marks indicate type I respiratory failure, and blue stars denote the onset of documented clinical improvement during subsequent antitumor treatment. The rightward arrows indicate ongoing treatment at the last follow-up. AE, adverse event; PD, progressive disease; PFS, progression-free survival; PLC, pulmonary lymphangitic carcinomatosis; PR, partial response; ToT, time on treatment (ToT was used for ensartinib in case 2 as treatment was discontinued because of toxicity without documented disease progression).

**Table 1:** Baseline clinical characteristics, molecular profiles, and treatment trajectories of the two patients.

**Parameter**	**Case 1**	**Case 2**
Age (years)/sex	52/female	49/female
Smoking history	Never	Never
Histology	Lung adenocarcinoma	Lung adenocarcinoma with intravascular tumor thrombus
Stage at diagnosis (AJCC 8th)	cT4N3M1c, IVB	cT3N1M1c, IVB
Metastatic sites at diagnosis	Extensive nodal, adrenal, hepatic, and multifocal osseous metastases	Multifocal osseous metastases
Brain metastasis	None	None
ALK detection method	Baseline: Ventana ALK (D5F3) CDx IHC; progression: plasma NGS	ADx-ARMS RT-PCR
ALK fusion/variant	*EML4–ALK* (E13:A20) variant 1	*ALK* fusion (variant not characterized)
First-line therapy	Ensartinib 225 mg QD (April 2024–November 2025)	Ensartinib (April–October 2025)
First-line duration/best response	18.9 months/PR	6.4 months/PR
Reason for switch	Disease progression with new low back pain and progressive osseous disease	Grade 3 constipation (intolerance)
Resistance NGS	*EML4–ALK* V1 + *ALK* p.L1196M (exon 23)	Not performed
Second-line therapy	Lorlatinib 100 mg QD (November 2025–April 2026)	Lorlatinib (November 2025–January 2026)
Second-line clinical course	Respiratory symptoms began approximately 3.5 months after lorlatinib initiation; clinically and radiologically suspected PLC and type I respiratory failure were documented in March–April 2026	Respiratory symptoms began approximately 1 month after lorlatinib initiation; clinically and radiologically suspected PLC and type I respiratory failure were documented in January 2026
Initial SpO_2_/O_2_ support at admission	92% on 4 L/min NC	96% on 2 L/min NC
Nadir SpO_2_/maximum O_2_ support during hospitalization	85%; escalated to HFNC	84%; escalated to HFNC
Admission ABG under supplemental oxygen (pH/PaO_2_/PaCO_2_/P:F)	7.42/57/41/154	7.46/63/35.7/121
Admission mMRC/ECOG	4/3	4/3
CRP (mg/L)	219	22.85
Third-line regimen	Iruplinalkib monotherapy (60 → 180 mg QD; initiated in April 2026)	Pemetrexed initiated in January 2026; iruplinalkib initiated 5 days later
Time to SpO_2_ improvement	Day 7	Day 7
Time to oxygen weaning/able to lie flat	Day 12/same day	Day 12/same day
Iruplinalkib-related adverse events	None	None
Current status	On treatment; PFS ongoing	On treatment; PFS ongoing

ABG, arterial blood gas; AJCC, American Joint Committee on Cancer; ALK, anaplastic lymphoma kinase; CRP, C-reactive protein; ECOG PS, Eastern Cooperative Oncology Group performance status; HFNC, high-flow nasal cannula; IHC, immunohistochemistry; mMRC, modified Medical Research Council dyspnea scale; NC, nasal cannula; NGS, next-generation sequencing; P:F ratio, PaO_2_/FiO_2_ ratio; PLC, pulmonary lymphangitic carcinomatosis; PR, partial response; PFS, progression-free survival; QD, quaque die (once daily); SpO_2_, peripheral oxygen saturation; FiO_2_, fraction of inspired oxygen; O_2_, oxygen; RT-PCR, real-time polymerase chain reaction.

### Case 1

#### Initial presentation and staging

A 52-year-old, never-smoking woman with no relevant occupational exposure, family history, or comorbidity was diagnosed with lung adenocarcinoma at an outside tertiary cancer center in April 2024. The disease was staged as cT4N3M1c stage IVB [American Joint Committee on Cancer (AJCC) Cancer Staging Manual 8th edition], with extensive nodal, adrenal, hepatic, and multifocal osseous metastases; brain MRI results showed no intracranial metastases. The ALK status on the diagnostic specimen determined by the Ventana ALK (D5F3) CDx immunohistochemistry (IHC) assay was positive; molecular profiling was not performed at diagnosis. The baseline levels of serum tumor markers measured in April 2024 were markedly elevated: CEA, 754 ng/mL; CYFRA21-1, 29.94 ng/mL; NSE, 51.96 ng/mL; and proGRP, 37.5 pg/mL. The baseline Eastern Cooperative Oncology Group (ECOG) performance status (PS) was 1.

#### Prior ALK-directed treatment

On the basis of the IHC-confirmed ALK rearrangement, first-line therapy with 225 mg of ensartinib once daily was initiated in April 2024, which produced a partial response (PR). Monthly incadronate disodium has been included since May 2025 as bone-targeted therapy. After approximately 19 months of disease control, the patient developed new-onset low back pain in November 2025. Subsequent clinical reassessment and imaging evaluation raised concerns for disease progression, particularly progression of osseous metastases. To clarify the underlying resistance mechanism and guide subsequent ALK-targeted treatment selection, peripheral blood next-generation sequencing (NGS; 229-gene panel) was performed in November 2025. This identified an EML4–ALK (E13) variant 1 fusion (allele frequency, 4.43%) together with an acquired ALK exon 23 point mutation, c.3586C > A (p.L1196M; allele frequency, 8.69%), which explains the on-target resistance to second-generation ALK inhibition. Following the molecular reassessment in November 2025, ensartinib was discontinued and second-line treatment with 100 mg of lorlatinib once daily was initiated.

#### Development and evaluation of clinically suspected PLC

Approximately 3.5 months later, the patient developed progressive exertional dyspnea, cough, and copious purulent sputum. A chest CT scan performed at a local hospital in March 2026 showed increased right-hilar soft tissue density and multiple bilateral patchy opacities, raising the possibility of PLC. Empirical antibiotic therapy (without corticosteroids) showed no improvement, and a follow-up CT scan in April 2026 was performed to document further progression. The patient was referred to our institution.

On admission, the patient was dyspneic at rest and unable to lie flat. The respiratory rate was 32 breaths/min, heart rate 110 beats/min, and SpO_2_ 92% on 4 L/min of supplemental oxygen via a nasal cannula, with bibasilar moist crackles (no velcro crackles), no palpable lymphadenopathy or hepatosplenomegaly, and mild bilateral lower-limb edema. ECOG PS was 3, and the modified Medical Research Council (mMRC) dyspnea grade was 4. Arterial blood gas (ABG) on supplemental oxygen showed a pH of 7.42, a PaO_2_ of 57 mmHg, a PaCO_2_ of 41 mmHg, and a PaO_2_/FiO_2_ ratio of 154, confirming type I respiratory failure. Inflammatory and tumor-burden markers were elevated [C-reactive protein (CRP), 219 mg/L; PCT, 0.14 ng/mL; IL-6, 38.42 pg/mL; D-dimer, 2.91 µg/mL; LDH, 219 U/L], whereas the NT-proBNP level was 186.52 pg/mL. In addition, a cardiac assessment showed no evidence of a cardiogenic component. Repeat serum tumor markers (on admission) are given as follows: CEA, 201 ng/mL; CYFRA21-1, 21.79 ng/mL; NSE, 47.91 ng/mL; proGRP, 65.4 pg/mL; AFP, 32.8 ng/mL; and SCC, 0.45 ng/mL. A contrast-enhanced chest CT scan (in April 2026) showed new diffuse bilateral patchy and reticular opacities with ill-defined margins, peribronchovascular interstitial thickening, mediastinal lymphadenopathy, and small bilateral pleural and pericardial effusions, which were accompanied by multiple rib and vertebral lesions and a left adrenal nodule. In the context of progressive metastatic lung adenocarcinoma, these findings were radiographically suggestive of PLC. However, pulmonary lymphatic involvement could not be confirmed via cytological or histopathological means (Figure 2A1, A2). Repeat tissue or plasma molecular profiling was not performed at disease progression when the patient was under lorlatinib treatment because of financial constraints, and therefore, the molecular resistance mechanism at that time remained unknown.

**Figure 2:**
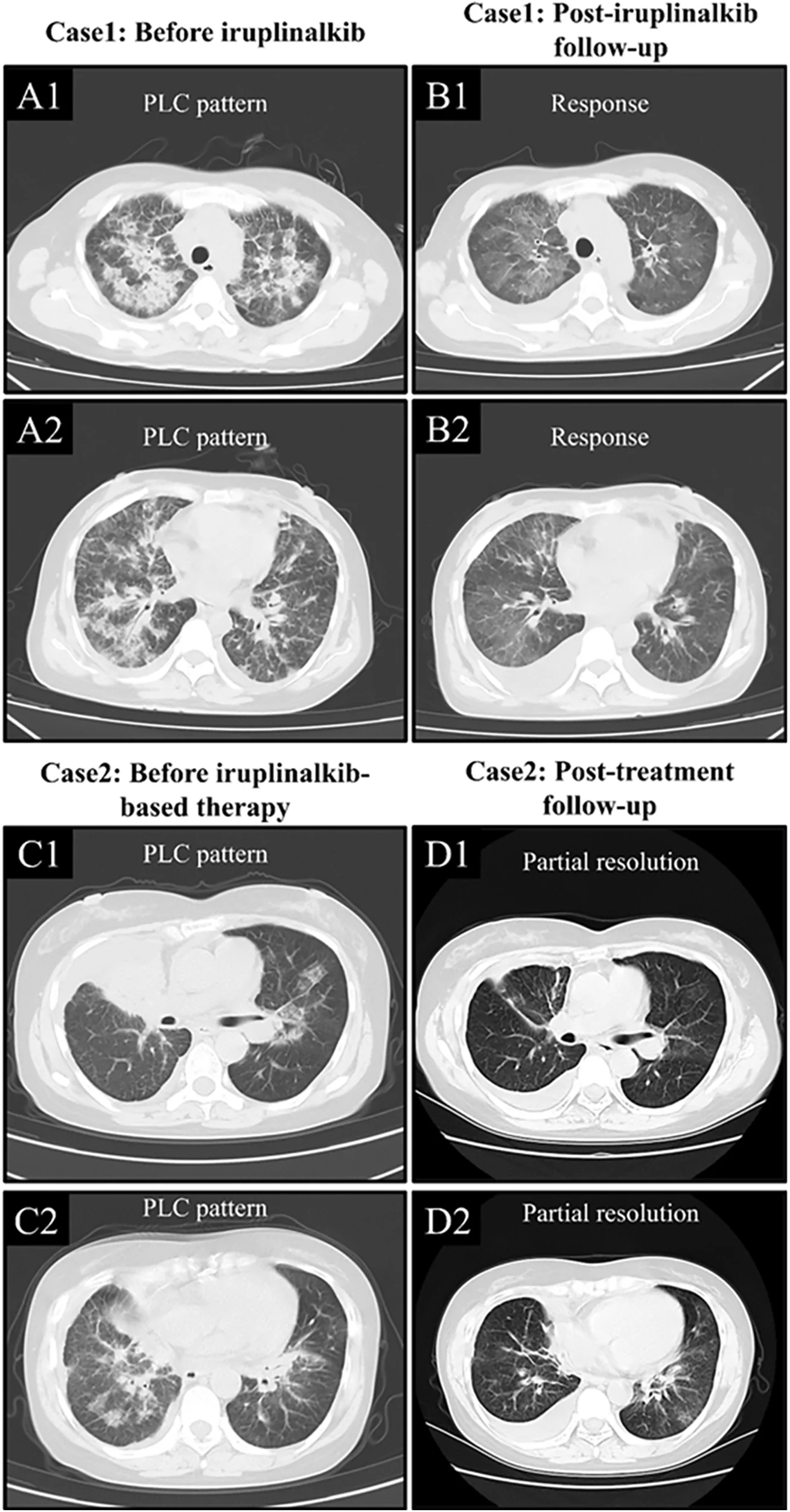
Serial chest CT scans showing pulmonary abnormalities clinically interpreted as suggestive of PLC and subsequent radiographic improvement during antitumor treatment. **(A1, A2)** CT images of case 1 showing diffuse bilateral peribronchovascular and reticular opacities suggestive of PLC at the onset of type I respiratory failure. **(B1, B2)** Follow-up CT images of case 1 showing marked radiographic improvement after iruplinalkib monotherapy. **(C1, C2)** CT images of case 2 showing progressive abnormalities suggestive of PLC with pleural effusion at the time of lorlatinib progression. **(D1, D2)** Follow-up CT images of case 2 showing partial resolution of the PLC pattern after iruplinalkib-based therapy combined with salvage pemetrexed.

Because the differential diagnosis of rapidly progressive bilateral opacities in this clinical context includes pulmonary infection, cardiogenic pulmonary edema, lorlatinib-induced interstitial lung disease, and malignant lymphangitic involvement, these possibilities were evaluated clinically. Bronchoscopy with bronchoalveolar lavage (BAL), performed primarily for microbiological evaluation, revealed scant airway secretions. BAL-fluid NGS detected rhinovirus C, whereas the results of sputum culture, BAL galactomannan, and serum (1,3)-β-D-glucan assays were negative; BAL cytology showed no malignant cells. Given the purulent sputum and the CRP level of 219 mg/L, concomitant infection could not be completely excluded. However, empirical antibacterial treatment produced no improvement in clinical or radiographic observation, and serial CT imaging results showed continued progression, arguing against infection as the sole explanation. Cardiogenic pulmonary edema was considered less likely because the NT-proBNP level was not markedly elevated and the available cardiac assessment showed no clinically significant cardiogenic component. Lorlatinib-induced interstitial lung disease was also considered less likely because lorlatinib withdrawal and corticosteroid treatment were ineffective, while the peribronchovascular and septal–reticular distribution, mediastinal lymphadenopathy, and pleural effusions favored malignant lymphangitic involvement. A transbronchial lung biopsy was not performed because severe hypoxemia (PaO_2_/FiO_2_ ratio, 154), tachypnea (32 breaths/min), orthopnea, and the inability to lie flat rendered the procedural risk unacceptable. Thus, despite the absence of pathological confirmation, progressive malignant lymphangitic involvement was considered the predominant explanation, and the condition was classified as “clinically and radiologically suspected PLC.”

#### Subsequent treatment and outcome

In view of the patient's life-threatening respiratory deterioration and the historical evidence of ALK-driven disease, iruplinalkib monotherapy was initiated on April 2026 at a dose of 60 mg once daily for a 7-day lead-in, followed by a dose of 180 mg once daily. Clinical improvement was rapid: SpO_2_ rose to 95%–98% by day 7, and supplemental oxygen was discontinued on day 12, the same day on which the patient was able to lie flat. Type I respiratory failure resolved, and ECOG PS improved to 2. Follow-up chest CT demonstrated radiographic improvement after iruplinalkib monotherapy (Figures 2B1, B2). The oxygenation trajectories of both patients are shown in Figure 3. No iruplinalkib-related adverse events of any grade, including hypertriglyceridemia, hypercholesterolemia, hepatic enzyme elevation, QTc prolongation, visual disturbance, or neurological symptoms, were observed. At the time of writing, the patient remains on iruplinalkib monotherapy with ongoing progression-free survival.

**Figure 3:**
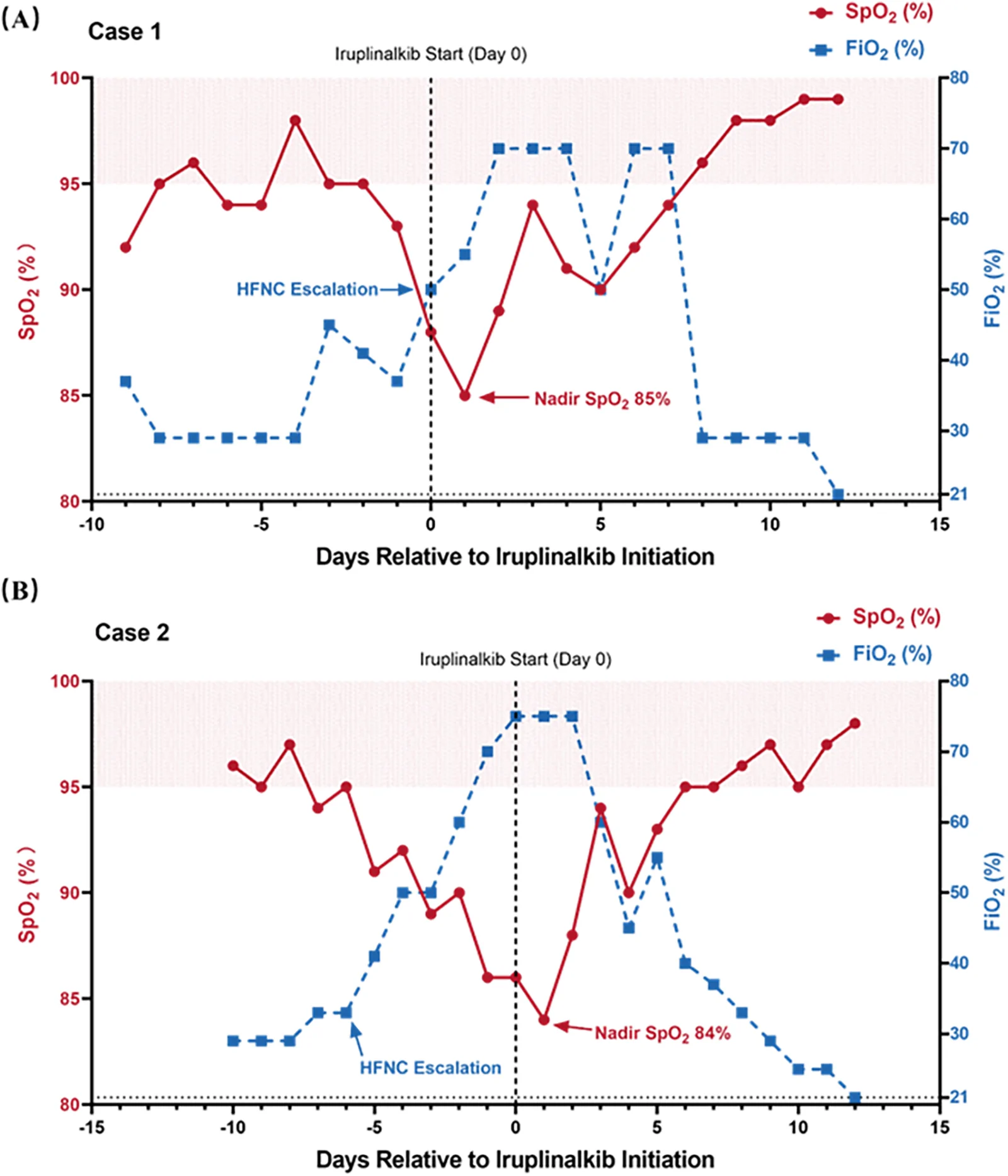
Dynamic monitoring of peripheral oxygen saturation (SpO₂) and fraction of inspired oxygen (FiO_2_) during the clinical course. **(A)** Respiratory trajectory of case 1. **(B)** Respiratory trajectory of case 2. The red solid lines indicate SpO_2_ levels, and blue dashed lines indicate the corresponding FiO_2_ requirements estimated from oxygen delivery settings. The gray shaded areas indicate the near-normal SpO_2_ reference range of 95%–100%. The vertical dashed lines denote initiation of iruplinalkib-based therapy. The arrows indicate escalation to high-flow nasal cannula (HFNC) oxygen therapy and the SpO_2_ nadir for each patient. After initiation of iruplinalkib-based therapy, both patients showed rapid improvement in SpO_2_ level and were weaned to room air (FiO_2_, 21%).

### Case 2

#### Initial presentation and staging

A 49-year-old never-smoking woman with no relevant occupational exposure, family history, or comorbidity presented to our emergency department on April 2025 with chest pain. A chest CT scan identified a right upper/middle lobe mass measuring approximately 47  mm × 38 mm, with lobulation, spiculation, pleural indentation, an air bronchogram, and spinous protrusion. A CT-guided lung biopsy demonstrated a poorly differentiated carcinoma consistent with lung adenocarcinoma, with conspicuous intravascular tumor thrombus identified within peritumoral lymphovascular spaces. The IHC results were positive for CK, CK7, TTF-1, and Napsin A (vimentin focally positive; CD34 highlighting vessels around the tumor thrombus) and negative for P40, CK5/6, PAX-8, GATA3, S-100, and P16, with retained INI1 expression. ALK fusion was detected via ADx-ARMS real-time PCR (the fusion partner and the variant could not be further characterized by this assay). Staging demonstrated multifocal osseous metastases without intracranial involvement. The disease was classified as cT3N1M1c, stage IVB (AJCC 8th edition). Baseline ECOG PS was 1, and serum tumor markers were within or near-normal limits (CEA, 2.49 ng/mL; CYFRA21-1, 3.72 ng/mL; NSE, 13.50 ng/mL; SCC, 0.56 ng/mL; proGRP, 38.7 pg/mL).

#### Prior ALK-directed treatment

First-line treatment with ensartinib was initiated on April 2025 and a PR was achieved that was sustained over 6 months. However, the patient developed Common Terminology Criteria for Adverse Events (CTCAE) grade 3 constipation that severely affected her daily life, which was caused by this intolerable toxicity rather than disease progression; ensartinib was discontinued on October 2025, and second-line treatment with lorlatinib was initiated on November 2025. Because the switch was driven by adverse events on a PR, repeat molecular profiling was not performed at this time.

#### Development and evaluation of clinically suspected PLC

Approximately 1 month after lorlatinib was started, the patient developed progressive cough, sputum production, and dyspnea and was subsequently readmitted to our institution on January 2026. On initial assessment, her respiratory rate was 30 breaths/minute and SpO_2_ was 96% while receiving 2 L/min of supplemental oxygen via the nasal cannula; however, her oxygen requirement increased during the early course of hospitalization. ECOG PS was 3, and the mMRC dyspnea grade was 4. An arterial blood gas analysis obtained under supplemental oxygen showed a pH of 7.46, a PaO_2_ of 63 mmHg, a PaCO_2_ of 35.7 mmHg, and a PaO_2_/FiO_2_ ratio of 121, confirming type I respiratory failure. Inflammatory markers were only modestly elevated, including a CRP level of 22.85 mg/L, a PCT level of 0.06 ng/mL, and an IL-6 level of 14.73 pg/mL, with a D-dimer level of 1.72 µg/mL, an NT-proBNP level of 69.22 pg/mL, and an LDH level of 191 U/L. A chest CT scan performed on January 2026 showed radiographic disease progression despite a relatively stable residual primary lesion. The new findings included diffuse bilateral patchy, ground-glass, and reticular opacities with ill-defined margins; thickening of the bronchovascular bundles; smooth interlobular septal thickening; and right pleural thickening, together with a new partly loculated right pleural effusion, increased pericardial effusion, and a newly visible right hepatic lobe nodule. The right upper lobe primary lesion measured 17 mm × 11 mm, smaller than the original baseline lesion of 47 mm × 38 mm, after the prior response to ensartinib and remained relatively unchanged on recent imaging, whereas the disseminated pulmonary opacities and effusions newly developed. The combination of peribronchovascular interstitial thickening, smooth interlobular septal thickening, progressive bilateral opacities, and a new pleural effusion was suggestive of PLC via radiographic findings in the context of metastatic lung adenocarcinoma (Figures 2C1, C2). However, bronchoscopy and lung biopsy were not performed because the patient had rapidly increasing oxygen requirements, a PaO_2_/FiO_2_ ratio of 121, a respiratory rate of 30 breaths/min, and an ECOG performance status of 3. Given the possibility of procedure-related worsening of hypoxemia and escalation to invasive respiratory support, the treating team judged that the procedural risks outweighed the expected diagnostic benefit. Consequently, pulmonary lymphatic involvement could not be cytologically or histopathologically confirmed. Repeat molecular profiling was not performed at this time because of financial constraints, and therefore, the resistance mechanism and the ALK fusion partner and variant could not be determined.

Because the differential diagnosis of rapidly progressive bilateral opacities in this clinical context includes pulmonary infection, cardiogenic pulmonary edema, lorlatinib-induced interstitial lung disease, and malignant lymphangitic involvement, these possibilities were evaluated clinically. Severe pulmonary infection was considered less likely because influenza PCR and sputum culture results were negative and the elevations in CRP and procalcitonin levels were modest, although infection could not be completely excluded. Cardiogenic pulmonary edema was not supported by the normal NT-proBNP level or the available cardiac and radiographic findings. Lorlatinib-induced interstitial lung disease remained an important consideration because respiratory symptoms developed approximately 1 month after the initiation of treatment; however, the absence of improvement after lorlatinib withdrawal and dexamethasone treatment, together with progressive peribronchovascular and smooth interlobular septal thickening, pleural involvement, and increasing pleural effusion, favored malignant lymphangitic involvement. Overall, clinically and radiologically suspected PLC was considered the predominant process, while the absence of pathological confirmation remained an important diagnostic limitation. The subsequent improvement during antitumor treatment retrospectively supported a predominantly malignant process.

#### Subsequent treatment and outcome

The addition of chemotherapy was not prompted by a specific clinical suspicion of loss of ALK dependence, and molecular reassessment was unavailable to evaluate this possibility. Rather, pemetrexed was added empirically because of the immediately life-threatening pulmonary disease burden and the need to broaden antitumor coverage after disease progression under lorlatinib treatment. Because the patient's severe hypoxemia, ECOG performance status of 3, and poor general condition raised substantial concerns regarding the tolerability of the guideline-recommended platinum–pemetrexed doublet (2, 3), platinum was omitted, and pemetrexed alone was selected as the chemotherapy component of the iruplinalkib-based regimen. Pemetrexed 800 mg was initiated on January 2026 and administered every 3 weeks for 6 cycles. Iruplinalkib was initiated 5 days after the first pemetrexed infusion, at a dose of 60 mg once daily for a 7-day lead-in, followed by a dose of 180 mg once daily. Grade 2 myelosuppression occurred after chemotherapy and was managed with a single dose of recombinant human granulocyte colony–stimulating factor. Respiratory improvement was rapid and followed a time course similar to that observed in case 1. The SpO_2_ level rose to 95%–98% by day 7 after iruplinalkib initiation; supplemental oxygen was discontinued on day 12, the same day on which the patient was able to lie flat, with full resolution of type I respiratory failure. Because pemetrexed was administered 5 days before iruplinalkib, the relative contributions of the two agents to the early clinical improvement could not be determined. The observed improvement may have reflected either treatment or their combined effect.

A follow-up chest CT imaging performed on March 2026 demonstrated partial absorption of the diffuse pulmonary abnormalities during the combination treatment. This radiographic improvement retrospectively supported a treatment-responsive malignant component but did not provide a pathological confirmation of PLC (Figures 2D1, D2). No adverse events attributable to iruplinalkib, including hypertriglyceridemia, hypercholesterolemia, hepatic enzyme elevation, QTc prolongation, visual disturbance, or neurological symptoms, were observed. At the time of writing, the patient remains on iruplinalkib-based therapy with sustained clinical benefit and ongoing follow-up. At present, neither plasma NGS nor a tissue rebiopsy is being planned, while the patient remains clinically stable on the current regimen, and financial constraints persist. Molecular reassessment has been deferred rather than definitively excluded and will be reconsidered at subsequent progression if it is expected to guide treatment and is financially feasible; a tissue rebiopsy would additionally require a safely accessible lesion and an acceptable procedural risk.

## Discussion

The two patients in this report developed severe hypoxemic respiratory deterioration with clinical and radiographic features of PLC after sequential treatment with ensartinib and lorlatinib. Rapid clinical and radiographic improvement was observed following the initiation of iruplinalkib monotherapy in case 1 and of iruplinalkib and pemetrexed in case 2. These observations extend the limited clinical experience with iruplinalkib-based treatment after lorlatinib exposure to a particularly urgent and underrepresented setting. The rapid improvement in oxygenation is clinically relevant because PLC may cause diffuse pulmonary involvement, rapidly increasing oxygen requirements, and life-threatening respiratory failure (11).

PLC is characterized by malignant involvement of the pulmonary lymphatic network and may be clinically suspected from an appropriate oncological background together with characteristic imaging findings (11, 17). In both patients, the diagnosis was made on clinical and radiological grounds. Serial CT imaging results demonstrated progressive peribronchovascular interstitial and interlobular septal thickening, bilateral pulmonary opacities, and pleural effusions—a pattern considered suggestive of lymphangitic tumor spread (17). Pulmonary infection, cardiogenic pulmonary edema, and lorlatinib-induced interstitial lung disease were evaluated as alternative diagnoses. Although a concomitant infectious component could not be completely excluded in case 1, these alternatives did not adequately explain the progressive radiographic abnormalities and respiratory deterioration. Pathological confirmation was not obtained because invasive lung biopsy was considered unsafe during severe hypoxemia.

Clinicoradiological studies have reported that ALK-rearranged NSCLC is more frequently associated with advanced nodal disease, pleural metastasis, pleural effusion, and lymphangitic carcinomatosis than ALK/EGFR-negative NSCLC (12, 13). Constitutive EML4–ALK signaling activates the RAS–MAPK, PI3K–AKT, and JAK–STAT pathways and may promote epithelial–mesenchymal transition, tumor cell migration, and invasion (18, 19). Lymphatic dissemination in NSCLC may also involve tumor-associated lymphangiogenesis, lymphatic endothelial remodeling, and VEGF-C/VEGFR-3 signaling (20). Although direct evidence linking ALK rearrangement to a specific lymphangiogenic program remains limited, these biological effects provide a plausible context for the aggressive lymphatic and pleural dissemination observed clinically (20). The lymphovascular tumor thrombus identified in the original biopsy of case 2 was consistent with an invasive phenotype, but this was not considered evidence of an ALK-specific mechanism of lymphatic spread.

Lorlatinib was developed through structure-based macrocyclic design to achieve potent ALK/ROS1 inhibition, broad activity against many single ALK resistance mutations, and enhanced central nervous system exposure (21, 22). Preclinical intracranial models, PET imaging in non-human primates, and clinical cerebrospinal fluid pharmacokinetic studies support its high blood–brain barrier permeability (22–24). These findings establish an important CNS exposure advantage but do not demonstrate preferential accumulation in the pulmonary interstitium or lymphatic vessels. Therefore, pulmonary progression during lorlatinib treatment cannot be attributed to inadequate pulmonary tissue exposure. In the absence of molecular reassessment, the mechanisms underlying progression during lorlatinib treatment in our patients remain unknown.

Iruplinalkib is a highly selective ALK/ROS1 tyrosine kinase inhibitor with reported preclinical activity against clinically relevant ALK resistance mutations, including L1196M and C1156Y (25). In case 1, plasma NGS at ensartinib progression identified EML4–ALK variant 1 (E13) together with ALK p.L1196M. Although this historical finding supported the clinical decision to maintain an ALK-directed treatment strategy, it could not define the resistance mechanism at subsequent progression during lorlatinib treatment. Because molecular reassessment was not performed at that time, the subsequent clinical improvement cannot be attributed specifically to the inhibition of L1196M. Evidence for the efficacy of iruplinalkib after disease progression under lorlatinib treatment remains limited. A small retrospective Chinese series reported an objective response rate of 27%, a disease control rate of 91%, and an estimated 12-month progression-free survival rate of 53% in patients treated with iruplinalkib after treatment with lorlatinib (10). A dedicated observational study is also ongoing (ClinicalTrials.gov identifier: NCT07374614). Unlike previously reported critical cases treated with crizotinib, alectinib, or brigatinib, our cases document treatment strategies incorporating iruplinalkib after sequential ensartinib and lorlatinib exposure, thereby extending this emerging clinical experience to patients with PLC-associated hypoxemic respiratory failure (14–16).

The outcome of case 2 should be interpreted in the context of combination treatment. Cytotoxic chemotherapy remains a guideline-supported systemic option after progression on multiple ALK TKIs (2, 3). Because the patient had severe hypoxemia, an ECOG performance status of 3, and rapidly progressive disease, pemetrexed was added to broaden antitumor coverage, whereas a platinum-containing doublet was considered unsuitable because of the patient's poor general condition. As pemetrexed was administered 5 days before treatment with iruplinalkib, its contribution cannot be separated from that of ALK-directed therapy, and the improvement in case 2 may have reflected either treatment or their combined effect. Pemetrexed-based chemotherapy has documented clinical activity in ALK-positive NSCLC (26, 27). In randomized trials in which single-agent pemetrexed was included as a chemotherapy comparator, radiographic assessments were scheduled every 6 weeks (28, 29). However, these protocol-defined assessment intervals do not establish the earliest onset of biological or symptomatic benefit and therefore do not exclude an earlier contribution from pemetrexed. Accordingly, the improvement in case 2 by day 7 cannot distinguish the effects of iruplinalkib from those of pemetrexed. Case 2 documents the clinical outcome of treatment with iruplinalkib and pemetrexed, whereas case 1 provides a complementary observation following iruplinalkib monotherapy.

This report has several limitations. First, it includes only two patients, precluding estimation of population-level efficacy, and the available follow-up does not allow firm conclusions regarding long-term survival. Second, neither patient underwent repeat tissue or plasma molecular profiling at disease progression under lorlatinib treatment, precluding direct characterization of the resistance mechanisms immediately before the initiation of iruplinalkib-based treatment. Third, PLC was diagnosed clinically and radiologically because severe hypoxemia made invasive pathological confirmation impractical. Moreover, a concomitant infectious component could not be completely excluded in case 1. Fourth, the concomitant administration of pemetrexed prevented treatment-specific attribution in case 2. Despite these limitations, the concordant and rapidly documented improvements in oxygenation, respiratory symptoms, and serial imaging provide clinically relevant experience regarding iruplinalkib-based management after lorlatinib exposure and support further prospective investigation in molecularly characterized patients.

## Conclusion

In summary, rapid clinical and radiographic improvement followed iruplinalkib monotherapy in case 1 and iruplinalkib plus pemetrexed in case 2. These observations extend the limited clinical experience with iruplinalkib-based treatment after lorlatinib exposure, although the contribution of pemetrexed in case 2 cannot be separated from that of iruplinalkib. Further prospective evaluation is warranted.

## Data Availability

The original contributions presented in the study are included in the article/supplementary material, and further inquiries can be directed to the corresponding authors.
